# Intralesional interferon alpha-2b as a novel treatment for periocular squamous cell carcinoma in horses

**DOI:** 10.1371/journal.pone.0297366

**Published:** 2024-02-21

**Authors:** Brittany B. Martabano, Steven Dow, Lyndah Chow, Margaret M. V. Williams, Maura K. Mack, Rebecca Bellone, Kathryn L. Wotman

**Affiliations:** 1 Department of Clinical Sciences, College of Veterinary Medicine and Biomedical Sciences, Colorado State University, Fort Collins, Colorado, United States of America; 2 Veterinary Genetics Laboratory and Department of Population Health and Reproduction, School of Veterinary Medicine, University of California-Davis, Davis, California, United States of America; Universidade Federal de Minas Gerais, BRAZIL

## Abstract

**Objective:**

To determine the safety and efficacy of perilesional human recombinant interferon alpha-2b (IFNα2b) for treatment of periocular squamous cell carcinoma (PSCC) in horses.

**Animals studied:**

Eleven horses (12 eyes) with PSCC were enrolled in this prospective clinical study with owner consent.

**Procedures:**

Systemically healthy horses were included in the study following confirmation of PSCC via biopsy. Every two weeks for a maximum of six treatments, horses were sedated and perilesional injection of IFNα2b (10 million IU) was performed. Tumors were measured prior to each injection and at one, three, and 12 months after treatment completion. A greater than 50% reduction in tumor size was considered positive response to treatment (i.e., partial or complete response). Development of anti-IFNα2b antibodies was assessed using serum samples obtained after treatment initiation and compared with treatment responses. Antibody concentrations were analyzed using a mixed model. Statistical significance was considered *p* < 0.05.

**Results:**

Each horse received four to six perilesional injections of IFNα2b. Five of 12 eyes (4/11 horses) responded to treatment. Two of five eyes showed complete resolution of gross PSCC. No systemic adverse effects were seen. Local swelling occurred during treatment protocol in 6/11 horses but resolved without intervention. All horses developed serum anti-IFNα2b antibodies. There was no evidence of statistical difference in antibody concentration between responders and non-responders.

**Conclusions:**

Perilesional administration of IFNα2b was found to be well-tolerated in horses with PSCC, and induced tumor regression in 42% of treated eyes. Treatment failure appears unrelated to the development of IFNα2b antibodies.

## Introduction

Squamous cell carcinoma (SCC) and periocular SCC (PSCC) are locally invasive tumors and the most common cancer of the equine eye [1]. Risk factors for the development of ocular or periocular SCC (PSCC) include lack of periocular skin pigmentation and exposure to high levels of solar radiation and ultraviolet light [2, 3]. Thoroughbreds, Haflingers, and draft horses also demonstrate an increased prevalence of SCC despite adequate ocular pigmentation [2–5], and a missense mutation in damage-specific DNA binding protein 2 (DDB2) has been discovered to be a causal risk factor for corneolimbal and third eyelid SCC in Haflinger and Belgian horses [6, 7].

There are a multitude of treatment modalities employed for treatment of PSCC with variable success [3, 4, 8–13]. Surgical excision of ocular SCC with appropriate margins and adjunctive therapy can be curative in some cases. However, surgical management of PSCC presents unique challenges due to the tight adhesion of the periocular skin to underlying fascia and bone [14]. Additionally, other treatment approaches such as radiation and photodynamic therapy may require general anesthesia or referral center care due to safety and equipment availability. Therefore, there is still a large unmet need for new approaches to management of PSCC, particularly those that can be delivered in an ambulatory setting.

Immunotherapy is now a well-established new modality for treatment for neoplasia in human medicine and relies on the activation of the patient immune system for elimination of neoplastic cells. In the case of SCC in humans, interferon alpha (IFNα) has a large body of research documenting its use as anti-cancer therapy [15]. Proposed mechanisms of action of IFNα treatment for SCC include down-regulation of tumor suppressor genes, upregulated tumor expression of major histocompatibility (MHC) class 1 molecules, activation of NK cells, and inhibition of tumor angiogenesis [16]. Numerous publications document the use of IFNα to enhance the cytotoxic effects of chemotherapies in the treatment of human head and neck SCC [17]. In one study, interferon alpha-2b (IFNα2b) was used successfully as both a topical and perilesional injection treatment for giant ocular surface SCC in human patients [18].

In the present study we investigated the safety and potential efficacy of the use of human recombinant IFNα2b as a novel treatment for equine PSCC. Clinical correlates of tumor responses to IFNa2b treatment were also assessed. A secondary aim was to evaluate treated horses for both the presence of the DDB2 missense mutation and for the development of serum antibodies to human recombinant IFNα2b and determine their influence on treatment outcome. We hypothesized that perilesional IFNα2b would induce tumor regression in treated horses with minimal side effects, that larger or more invasive lesions would be less likely to respond, and that homozygosity for the DDB missense variant and/or anti-IFNα2b serum antibodies would be associated with lack of treatment responses.

## Materials and methods

### Case inclusion

The study was approved by the Colorado State University Clinical Review Board (protocol VCS#2018–164). Horses presenting to the Colorado State University Veterinary Teaching Hospital with suspect PSCC were considered for enrollment if they were found to be systemically healthy based on physical examination, complete blood count, serum biochemistry, and measurement of quantitative fibrinogen. Complete ophthalmic examinations were performed on each horse by a single investigator (KLW) after intravenous (IV) sedation with detomidine hydrochloride (Dormosedan^®^ 0.01–0.02 mg/kg, Pfizer Animal Health, Exton, Pennsylvania, USA) and auriculopalpebral nerve block (0.5ml of 2% lidocaine (Vet One, Boise, Idaho)) prior to slit-lamp biomicroscopy (Kowa SL-15, Kowa, Toyko, Japan), direct ophthalmoscopy (Welch Allen direct ophthalmoscope, Welch Allyn Distributors, Skaneateles Falls, NY), fluorescein stain (fluorescein sodium ophthalmic strips, OptiTech Eyecare, Allahabad, India), rebound tonometry (TonoVet^®^, Jorgensen Distributors, Fort Collins, CO), and digital palpation of the upper and lower conjunctival fornix and third eyelid. Suspect PSCC lesions were biopsied and sent for histopathologic analysis through the Colorado State University Diagnostic Lab. If histopathology confirmed SCC with tumor localized to the eyelid, horses were enrolled after written owner consent. Horses were excluded if lesions of the cornea, limbus, bulbar conjunctiva, or third eyelid were noted. Owners were advised that the IFNα2b protocol was experimental and additional therapeutic options were reviewed prior to study enrollment.

### Treatment procedures

Treatment with IFNα2b consisted of perilesional injections performed at two week intervals for a minimum of four and a total of up to six treatments. The total number of injections was determined at the discretion of the lead investigator (KLW) and based on integrity of the tissue to be injected and response to prior treatments.

At each treatment, horses were sedated with IV detomidine hydrochloride for complete ophthalmic examination as described previously, photographs of the affected eye(s) (Nikon D160 DSLR, micro Nikkor 105mm lens, Nikon Inc., Melville, NY), and perilesional IFNα2b injection. In preparation for the injection, the periocular skin and conjunctival fornix was cleaned with dilute betadine solution (1:50) and prepared with topical 2% lidocaine gel and 0.5% proparacaine (Akten™, Akorn Inc., Lake Forest, IL and Alcaine™, Alcon Laboratories, Inc. Fort Worth, TX), respectively. Human recombinant IFNα2b (Intron^®^A powder for injection, 10 million IU per vial Schering Corporation, Kenilworth, NJ) was sterilely reconstituted following manufacturer’s instructions with 1 ml of diluent added to one vial of powdered IFNα2b and resulted in a total reconstituted volume of 1.2 ml, the entirety of which was used at each injection regardless of lesion size. All perilesional injections were performed sterilely by one investigator (KLW) using a tuberculin syringe with 27-gauge needle. The injection was directed dorsal to the lesion when possible, carefully avoiding necrotic or friable tissue to prevent leakage of the drug from the skin. Drug was evenly distributed between lesions when applicable. Topical antibiotic ointment (Neomycin, Polymyxin B Sulfates, Bacitracin Zinc Ophthalmic Ointment U.S.P., Bausch & Lomb Pharmaceuticals, Inc., Tampa, FL) was applied over the tumor and injection sites immediately following the procedure. Horses were monitored for local or systemic reactions for four hours following each injection and lesions were photographed prior to dismissal. Heart rate, respiratory rate, and rectal temperature were recorded every hour for three hours following sedation and injection. Horses were discharged from the hospital and topical antibiotic ointment was applied by the owner twice daily thereafter for one week.

Blood was drawn and quantitative fibrinogen was measured prior to the first injection (baseline), four hours post-injection, and just prior to the second injection.

### Monitoring and follow up

At each visit, prior to IFNα2b injection, lesions were photographed with a ruler in the frame. Image J software (https://imagej.nih.gov/ij/) was used to analyze tumor size (cm^2^). Approximately one and three months after the final IFNα2b injection, horses returned for follow-up examinations during which a full ophthalmic examination was performed and ophthalmic photographs were obtained. Follow-up at one year after the final injection was performed via a combination of phone interviews with owners, photographs, and ophthalmic examinations. Tumors were classified using a system based on the World Health Organization criteria for tumor response guidelines for measurable lesions [19]. Broadly, tumors were considered responders to treatment if they fell into the classification of complete response (disappearance of entire lesion) or partial response (at least 50% decrease from baseline measurement) and non-responders if they fell into the classification of progressive disease (at least a 25% increase from baseline measurement) or stable disease (insufficient change to be considered progressive or responding).

### Genetic analysis

For each horse, whole blood and approximately 50 mane hairs with the root bulb were collected. Genomic DNA was isolated from either whole blood or hair samples using a DNA extraction kit (Puregene, Qiagen Inc., Valencia, CA, USA) and previously published protocols. Following extraction, genotyping for the DDB2 c.1013 C>T risk variant was performed at the UC Davis Veterinary Genetics Laboratory using a commercially available assay. Appropriate controls, including positive controls for each genotype and a negative control (no DNA) were performed with each assay.

### Anti-interferon serum antibody analysis

Blood samples were collected from each horse for anti-IFNα2b serum antibodies prior to the second, third, and fourth IFNα2b injections (two, four, and six weeks post treatment initiation) and serum was frozen at -80C until the analysis was performed. If further injections were performed, blood was also collected for anti-IFNα2b antibodies at later time points. A 96-well ELISA plate (Thermo Fisher Scientific, Waltham, MA) was coated with 100μl of human interferon alpha-2b (Intron^®^A powder for injection, Schering Corporation, Kenilworth, NJ) diluted in 0.1 M Sodium Bicarbonate to a concentration of 1μg/mL and incubated overnight at room temperature. The plate was washed with ELISA wash buffer (PBS + 0.05% Tween20, Sigma-Aldrich, Inc. St. Louis, MO) three times and 200 μl of 5% Non-fat dry milk (Sigma-Aldrich) was used to block each well. The plate was allowed to incubate at room temperature for 1 hour. After washing the microplate with ELISA wash buffer three times again, diluted horse serum samples were added to the wells in triplicates. Horse serum samples were diluted in 3% BSA in PBS at a dilution of 1:2500. The plate was incubated at room temperature for an hour. Following another three washes with ELISA wash buffer, 100μl of Peroxidase Affinipure Goat Anti-horse IgG (Code: 108-035-003, Jackson Immunoresearch Laboratories, Inc, West Grove, PA) diluted 1:5000 in 3% BSA in PBS was added to each well and the plate was incubated at room temperature for another hour. The plate was washed three more times and 100μl of TMB substrate solution (Thermo Fisher Scientific) was added to each well. Out of direct light, the plate incubated for 5 minutes at room temperature before 50μl of 0.16M sulfuric acid was added to each well to stop the reaction. The optical density of each well was read at 450nm on a Biotek Synergy HT microplate reader. Normal horse serum was used as a control. Anti-IFNα2b serum antibody concentration was measured in triplicate and the mean value was reported at each time point.

### Statistical analysis

Mean ± standard deviation (s.d.) anti-IFNα2b serum antibody concentrations at each time point were compared between responders and non-responders for samples taken two, four, and six weeks after the initiation of IFNα2b treatment. Residual diagnostic plots of the data were assessed for normality. A mixed model was fit using anti-IFNα2b serum antibody concentration as the response. Group (responder or non-responder), week (two, four, or six), and group*week interaction were included as fixed effects. Horse was included as a random effect accounting for the repeated measurements over time. Mean ± s.d. quantitative fibrinogen levels were compared using one-way analysis of variance (ANOVA). Evidence of a statistical difference was considered *p* < 0.05.

## Results

### Study population

Eleven horses (eight geldings and three mares, aged 14.5 ± 3.8 years, [mean ± s.d.]) and a total of 12 eyes with PSCC were treated with perilesional IFNα2b as a part of this pilot study. All horses received between four and six treatments. No horses had a history of ongoing systemic illness nor any evidence of systemic abnormalities on physical examination or bloodwork. Four horses had been treated for PSCC prior to enrollment in the study. Case 3 was treated with surgical excision two years prior and was enrolled following recurrence of the mass. Cases 4 and 5 were treated with debulking and placement of cisplatin beads three and 17 months prior to enrollment, respectively. Three years prior to enrollment in the study, Case 11 was treated with topical chemotherapy (unknown type) with no response after three weeks.

### Tumor response to treatment

**Fig 1:**
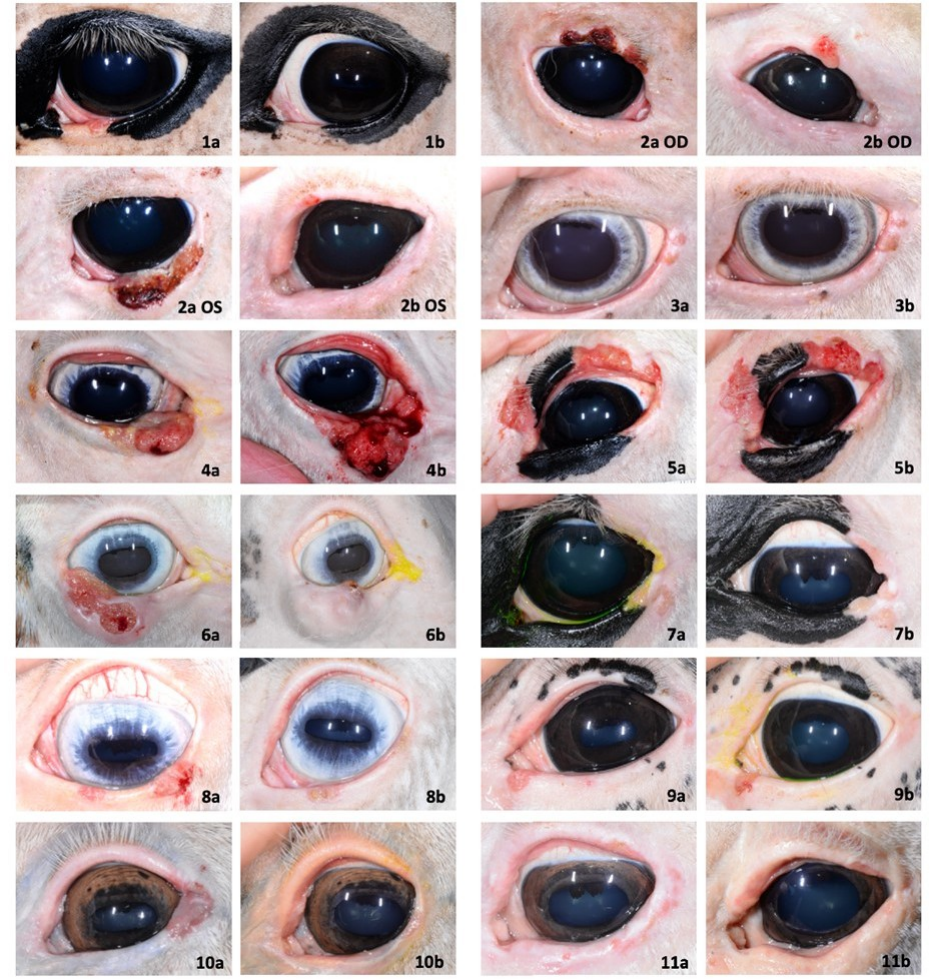
Clinical composite of the 11 study horses with biopsy confirmed periocular squamous cell carcinoma (PSCC). Each horse received perilesional injections of IFNα2b for PSCC every two weeks starting at time 0 for a total of four to six injections. Responders had greater than 50% reduction in tumor size after completion of treatment. Pre- and post-perilesional IFNα2b treatment photos taken at each patient’s initial ophthalmic examination (a) and last follow-up examination (b).

Tumors in 5 of 12 eyes (4/11 horses) were considered responders to treatment by perilesional injection of IFNα2b. A summary of each case including signalment, tumor location, tumor size pre- and post-treatment, number of injections, local reaction incidence and follow-up or additional intervention performed can be found in Table 1, and composite images of each case are shown in Fig 1.

**Table 1:** Study population data, response to treatment, number of injections, incidence of local reactions, follow-up, additional intervention data for 11 horses (12 eyes) receiving perilesional injections of IFNα2b for periocular squamous cell carcinoma (PSCC) tumors every two weeks starting at time 0 for a total of four to six injections.

Case	Signalment (age at initial presentation)	Tumor location	Pre-treatment tumor size (cm^2^)	Post-treatment tumor size (cm^2^) at last follow-up	Percentage change in tumor at last follow-up	# injections	Local reaction	Follow-up/additional intervention
1	7 y.o. American Paint gelding	Inferior nasal eyelid OS	3.05	0	Responder—complete resolution	4	N	Disease free at _ months (owner photographs)
2	13 y.o. American Paint gelding	Superior eyelid OD, inferior eyelid OS	OD– 12.9 OS– 26.4	OD– 3.3 OS—0	Responder OD– 74% reduction (partial response) OS–complete resolution	OD– 6 OS -6	Y; OS inj. 4	Surgical resection OD, Disease free OU at _ months (owner photographs)
3	12 y.o. Tennessee Walker gelding	Inferior lateral eyelid OS	1.71	2.23	Non-responder– 18% increase (stable disease)	4	Y; inj. 4	Surgical resection
4	15 y.o. American Paint gelding	Inferior eyelid OD	22.5	27.6	Non-responder– 23% increase (stable disease)	3	N	Owner withdrawn following inj. 3, enucleated
5	20 y.o. American Paint gelding	Superior eyelid OS	30.4	29.4	Non-responder– 3% reduction (stable disease)	5	N	Lost to follow up
6	17 y.o. American Paint mare	Inferior eyelid OD	41.3	54.6	Non-responder– 32% increase (progressive disease)	4	N	Enucleated by rDVM 13 months post-enrollment
7	17 y.o. American Paint gelding	Inferior lateral eyelid OS	5.6	10.0	Non-responder– 80% increase (progressive disease)	4	N	Lost to follow up
8	10 y.o. American Paint mare	Inferior eyelid OS	7.6	3.7	Responder– 51% reduction (partial response)	4	Y; inj. 4	Surgery not pursued by owner
9	15 y.o. Pony of America gelding	Inferior medial eyelid OS	2.7	2.1	Non-responder– 19% reduction (stable disease)	5	Y; inj. 3	Surgical resection
10	19 y.o. American Paint gelding	Inferior lateral eyelid OS	10.2	1.7	Responder– 83% reduction (partial response)	5	Y; inj. 5	Surgical resection
11	15 y.o. American Paint mare	Lateral canthus OS	2.0	2.1	Non-responder– 5% increase (stable disease)	4	Y; inj. 2	Stable disease at 2 year follow up

No horses showed any evidence of systemic reaction during the 3-hour post-injection monitoring period. Six of 11 horses experienced local swelling at the injection site once during their treatment protocol. All local reactions resolved within 24 hours without the addition of systemic anti-inflammatory medication.

### DDB2 mutation status and treatment responses

All horses tested homozygous for the reference allele for the DDB2 genetic risk variant (c.1013 C>). Thus no evaluation on impact of this genetic variant and treatment response could be performed.

### Fibrinogen concentrations and correlation with treatment responses

There was no evidence of a statistically significant difference between responders and non-responders when comparing quantitative fibrinogen concentrations at baseline, four hours after injection one, or prior to the second injection.

### Development of anti-IFNα2b serum antibody responses and treatment responses

**Fig 2:**
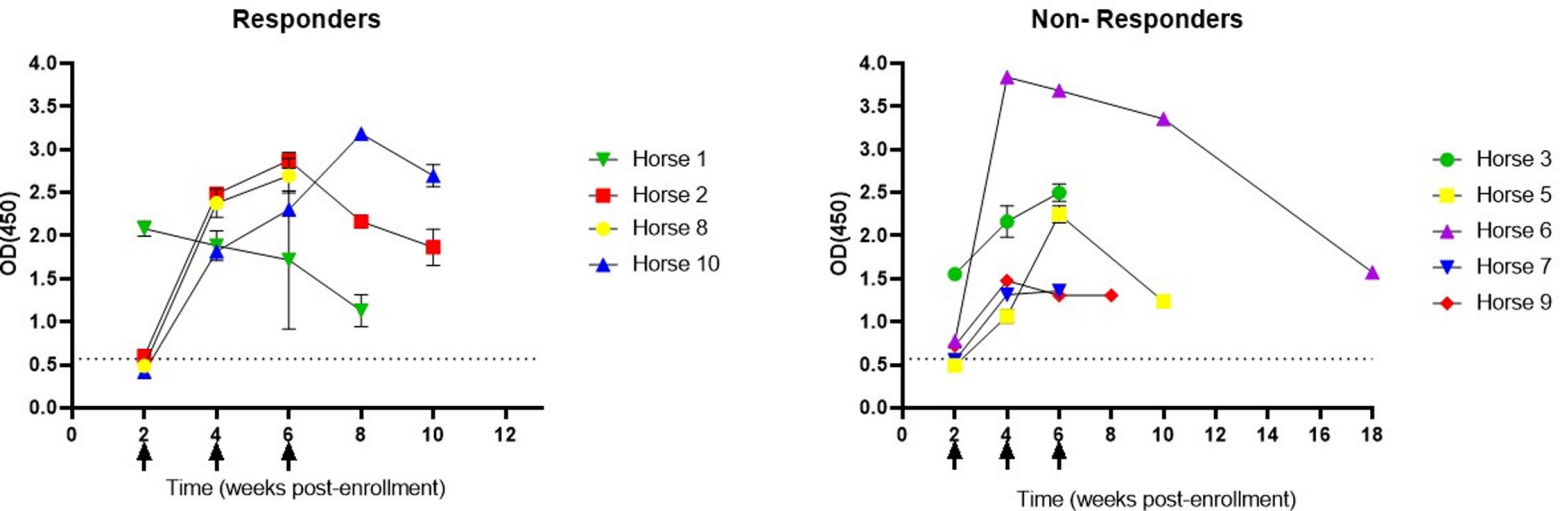
Optical density (OD) at 450 nm measurement of serum antibodies to interferon α2b in 11 horses (each represented by a different color in the line graphs) receiving perilesional injections of human recombinant IFNα2b for periocular squamous cell carcinoma tumors every two weeks starting at time 0 for a total of four to six injections. Responders had greater than 50% reduction in tumor size after completion of treatment. Each arrow along the x axis represents the time of perilesional treatment and the mean value of untreated normal serum is represented with dotted line. No significant difference in mean ± standard deviation IFNα2b serum antibodies was found between responders and non-responders at two, four, or six week post treatment initiation. Later time points included insufficient data for comparison.

All horses treated with IFNα2b developed antibodies to the protein during treatment. There was no evidence of a statistical difference in IFNα2b serum antibody concentrations between responders and non-responders. Unfortunately, pre-treatment serum samples were not available for analysis in this study, but we evaluated serum samples from three clinically normal horses (without PSCC) to provide a baseline for anti-IFN antibody responses, and none of the healthy control horses had detectable antibodies to IFNα2b. A graphical representation of IFNα2b serum antibody concentrations is provided in Fig 2.

## Discussion

Key findings from this study were that repeated perilesional human recombinant IFNα2b injections were well-tolerated and resulted in a 42% response rate in equine eyes with PSCC, with some tumors showing complete regression. This study therefore provides the first clinical evidence that human recombinant IFNα2b can be used successfully to treat PSCC in horses.

All treated horses developed serum antibodies to IFNα2b, but there was no evidence of a difference between antibody development in responders *versus* non-responders. Human studies have documented in vivo production of IFN antibodies following treatment for a variety of conditions including hepatitis C [20, 21], multiple sclerosis [22], midgut carcinoid tumors [23], and hairy cell leukemia [24]. Production of IFN antibodies interferes with IFN activity against its receptor target and has been shown to cause decreased treatment efficacy and/or IFN treatment failure in human patients with SCC [25]. The fact that production of serum antibodies to IFNα2b did not appear to affect tumor response in this study could be because antibody concentration is not related to treatment outcome in equine PSCC, or because the number of horses in this study is insufficient to demonstrate a difference between responders (n = 4 horses, 5 tumors) and non-responders (n = 7 horses, 7 tumors).

There was no trend toward size of lesion or location of a lesion being more responsive to treatment. One of the largest lesions (26.4 cm^2^) and one of the smallest lesions (3.05 cm^2^) were those that responded best to treatment. These were also the first two cases enrolled in the study, therefore experience with the procedure did not necessarily yield better outcomes over time. Case 1 was debulked via the biopsy prior to treatment, but Case 2 (OU) was too large and erosive to perform any form of productive debulking and yet both yielded the most profound outcomes. In contrast, Case 9 was debulked in a manner similar to Case 1 and showed no significant response to treatment. It is acknowledged that a larger sample size may have resulted in more quantifiable trends.

It is difficult to directly compare these results to other studies of PSCC management in horses, as most of the prior studies involve surgical excision of gross tumor prior to the treatment of interest, whereas only three tumors in our study had been previously surgically debulked. Previous studies have evaluated surgical excision alone and in combination with multiple adjunctive modalities including 90Sr beta irradiation, cryotherapy, radiofrequency, Cesium 137 interstitial radiography, an SCC vaccine, and photodynamic therapy [8, 14, 26]. Because gross disease was removed prior to the treatment under investigation, these studies all evaluated time to recurrence. In the current study, we chose to evaluate response to perilesional IFNα2b alone, without surgical excision, to determine if this would be a viable treatment alternative in a setting where surgical excision could not first be performed (e.g., a client unable without access to a referral facility, in an ambulatory setting, or a tumor too difficult to resect). Outcomes may have differed if surgical excision followed by infiltration of the surgical margins with IFNα2b and monitoring of disease recurrence had been studied.

While many of the tumors in this study were considered non-responders to treatment, two tumors experienced complete regression and three tumors underwent partial regression. In addition, five of seven tumors considered non-responders met the WHO classification of stable disease and only two were considered to have progressive disease. Thus, perilesional injection of IFNa2b holds considerable promise for managing equine PSCC, as the biological response rate (tumors showing complete resolution, partial resolution, or stable disease) in this study was 83%.

These findings indicate that IFNα2b could be used as an easily performed, first-line treatment, with the potential to yield complete tumor resolution or significant reduction in tumor size in a case that may have otherwise required complicated eyelid reconstruction or enucleation. Case 2 OD showed marked improvement in the size of the tumor, allowing for routine standing wedge resection with clean margins, a procedure that would not have been feasible prior to IFNα2b treatment due to the extent of the lesion. Additionally, IFNα2b has key advantages over other therapies in that it is readily obtained from pharmacies, relatively inexpensive, safe to handle, and straightforward to inject.

Study limitations include the small sample size (n = 11 horses, 12 tumors) and the relatively short follow-up time with which to assess tumor recurrence. In addition, none of the study horses had the DDB2 variant associated with PSCC risk in certain breeds of horses. Therefore, testing in horses with varying genotypes is needed to determine if this locus impacts response to treatment and could inform precision medicine approaches to treat cancer in horses.- Due to owner limitations or lack of compliance not all horses that fit the study inclusion criteria returned for all the designated follow up appointments. Horses were examined in person for follow up examinations when possible, but some were only evaluated via owner photos and others were lost to follow-up. A larger number of horses with more consistent follow up evaluations would have provided valuable data. Additionally, because this study also focused on the safety of IFNα2b as a perilesional injection in horses, one selected dose of medication was used regardless of tumor size. It is possible that different dosing schemes could have yielded different results.

## Conclusions

Perilesional injections of human recombinant IFNα2b was found to be a safe and effective new approach to management of PSCC in horses. Moreover, IFNα2b injections could be readily combined with other treatment modalities, including surgical excision, local radiation therapy, and local cyotoxic chemotherapy to improve response rates. This new local immunotherapy can be readily applied to treatment of PSCC in horses in a setting that does not require general anesthesia or hospitalization. Thus, the clinical impact of this treatment may be substantial in horses.
